# A case of non‐cardiogenic pulmonary edema provoked by intravenous acetazolamide

**DOI:** 10.1002/ams2.279

**Published:** 2017-04-24

**Authors:** Yuichiro Ono, Makiko Morifusa, Satoru Ikeda, Chika Kunishige, Yoshiki Tohma

**Affiliations:** ^1^ Hyogo Prefectural Kakogawa Medical Center Kakogawa Hyogo Japan

**Keywords:** acetazolamide, anaphylaxis, drug side‐effects, pulmonary edema, respiratory insufficiency

## Abstract

**Case:**

A 61‐year‐old man was diagnosed with severe chest trauma after a car accident and had had difficulty in weaning from a ventilator because of flail chest and dilated cardiomyopathy. On the 17th day in the intensive care unit, he received i.v. acetazolamide to increase urine output. One hour after the injection, he suddenly developed severe hypoxia. Chest radiography revealed a butterfly shadow. He received other diuretics and a vasodilator, which seemed slowly to resolve the respiratory failure. Five days later, acetazolamide was again given and he experienced the same deterioration.

**Outcome:**

We concluded that the episodes were attributed to pulmonary edema provoked by acetazolamide.

**Conclusion:**

Acute non‐cardiogenic pulmonary edema is an uncommon and lethal adverse effect of acetazolamide. Careful attention may be warranted when administering acetazolamide to critically ill patients.

## Background

Acetazolamide is a carbonic anhydrase inhibitor (CAI) that is widely applied in various clinical settings. This drug inhibits the conversion of carbon dioxide and water into carbonic acid, and has been used to treat high intraocular pressure and high altitude disease, to increase urine volume, and correct metabolic alkalosis. Although CAIs are frequently used, acute non‐cardiogenic pulmonary edema has not been well‐recognized as one of its cryptogenic and fatal adverse effects. We report our experience with a patient who encountered two episodes of pulmonary edema after receiving acetazolamide.

## Case

A 61‐year‐old man had been admitted to an intensive care unit due to severe blunt chest wall trauma from a motor vehicle accident. His injuries were right massive hemopneumothorax, a flail chest and intrahepatic hematoma. Two thoracic catheters were inserted into his right thorax, and computed tomography revealed active bleeding from three vessels, which was controlled by transcatheter arterial embolization. The patient had required prolonged ventilator support owing to his chest wall instability and idiopathic dilated cardiomyopathy with an ejection fraction of approximately 30%, which had been previously evaluated. We had trouble liberating him from a ventilator and carried out tracheostomy on the 10th day of hospitalization.

**Figure 1:**
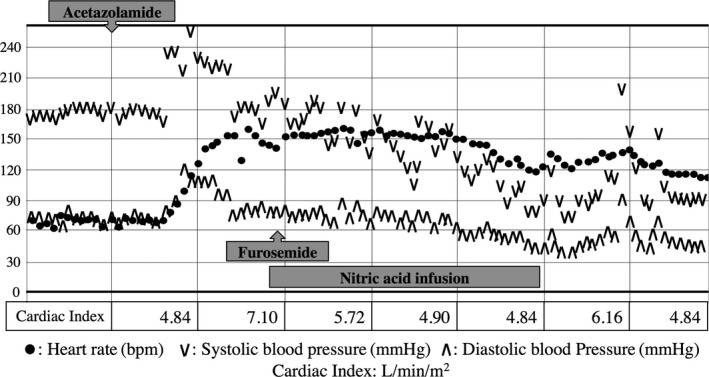
Five‐minute trends of heart rate and blood pressure in a 61‐year‐old man with severe chest trauma treated with i.v. acetazolamide. Approximately 1 h after acetazolamide infusion, both heart rate and blood pressure increased remarkably. The condition continued for approximately 8 h then gradually resolved. Cardiac indexes (bottom line) measured with a radial arterial catheter suggested the patient's hyperdynamic state.

**Figure 2:**
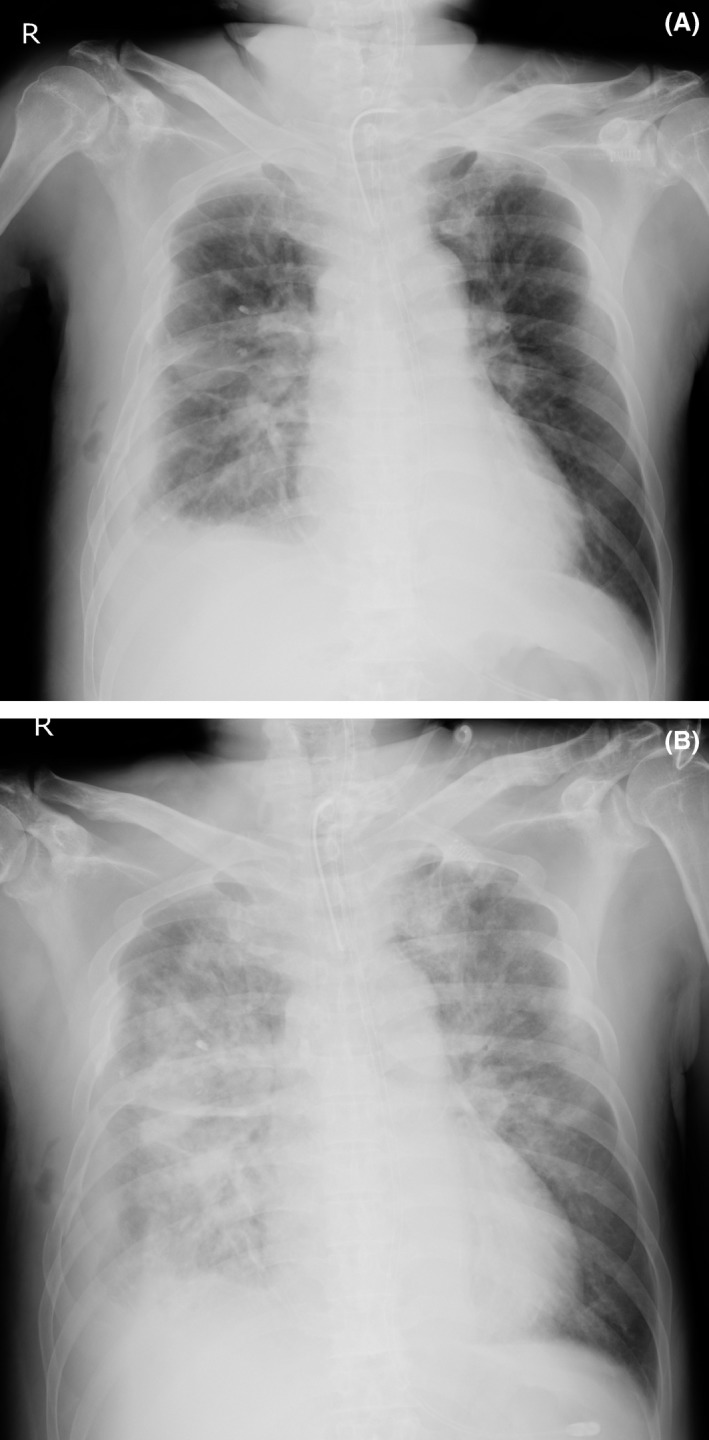
Chest X‐rays of a 61‐year‐old man with severe chest trauma treated with i.v. acetazolamide. A, Chest X‐ray routinely taken on the morning of the first event day. It shows pleural effusion, a vanishing tumor, multiple costal fractures, and subcutaneous emphysema on the right chest. B, Chest X‐ray taken during the attack. Additional findings, such as a bilateral butterfly shadow and air bronchograms, are present.

On the 17th day, the patient was still receiving ventilator support. Thus, in order to increase urine output and correct metabolic alkalosis, 500 mg acetazolamide was given i.v. One hour after the injection, he suddenly developed hypertension, tachycardia (Fig. 1), and hypoxemia (PaO_2_/FiO_2_ ratio was less than 100 mmHg). Wheezing sounds were heard on chest auscultation and much pinkish foamy secretion was suctioned through his tracheal tube. He showed choking‐like signs and his ventilator monitor showed an extremely high airway pressure and significantly decreased tidal volume, which limited its function, and he was transitioned to manual ventilation. Chest radiography (Fig. 2) revealed a bilateral butterfly shadow. We assumed that he developed acute pulmonary edema caused by acute exacerbation of congestive heart failure. Therefore, the respiratory failure was treated using diuretics, nitric acid, and an inhaled β_2_ stimulant to help relieve the airway pressure. As the event resolved after approximately 8 h, we did not advance a detailed evaluation.

However, 5 days following the previous episode, the same dose of acetazolamide was again given for the same purpose, and the patient experienced an abrupt episode of respiratory failure that was identical to the first. Over the period of the second episode, we managed to carry out additional evaluations, other than chest radiography. A ventilator recorded extremely poor lung compliance, which was less than 10 mL/cmH_2_O, and slightly increased airway resistance. These suggested the extremely high airway pressure was not derived from bronchospasm, but from pulmonary parenchymal edema. Moreover, echocardiography revealed a hypercontractile left ventricle compared to the usual state, although there were no signs or evidence of significant fluid overload. Only β‐blocker infusion was selected to suppress catecholamine surge‐like symptoms, such as hypertension and tachycardia, and the second episode spontaneously resolved as well.

In the two episodes, the symptoms were provoked just 1 h after the acetazolamide shot, but there were no apparent signs or evidence of anaphylaxis, such as bronchospasm, cutaneous and mucosal lesions, or shock. Although dilated cardiomyopathy might affect respiratory failure, the patient's left ventricular contraction observed by echocardiogram relatively improved during the episodes. In addition, the cardiac indexes measured by a radial arterial catheter, as described in Figure 1, suggested that the patient cardiac output had increased rather than decreased during the attack. Therefore, it was difficult to attribute the pathophysiology as purely cardiogenic. Finally, we concluded that the pulmonary edema was provoked by acetazolamide. We subsequently avoided the use of acetazolamide and the patient did not experience any further episodes of severe respiratory deterioration, which suggested that the episodes could be associated to the drug.

## Discussion

Non‐cardiogenic pulmonary edema triggered by a carbonic anhydrase inhibitor is a very rare adverse effect. There are few case reports regarding this topic;^1^, ^2^, ^3^, ^4^, ^5^ interestingly, all of them relate to the drug's ophthalmological purpose. Furthermore, no formal case reports have described this adverse effect in critically ill patients, despite its frequent appearance in intensive care units as well as Japan. However, clinical guidelines for the appropriate use of CAIs^6^ published in 2015 by four Japanese medical societies clearly warn the risk of respiratory failure and death in some cases. The guidelines briefly cover several critical cases that were identical to ours.

Although the mechanism for this complication has been idiosyncratic and unexplained, some consideration is given. Acetazolamide is classified into a sulfonamide group for its structure. It has been known that the group, for example, hydrochlorothiazide and sulfamethoxazole, causes non‐cardiogenic pulmonary edema.^7^ Hence, the pathophysiology is thought to be associated with both sulfonamide cross‐sensitivity and its immunomediated mechanism,^1^, ^3^ which could increase the capillary endothelium permeability leading protein and fluid to enter the lung parenchyma and alveolar spaces.^8^ Bernal *et al*.^9^ found that one patient suffering from hydrochlorothiazide‐induced non‐cardiogenic pulmonary edema had decreased serum immunoglobulin G and suggested that drug‐induced immunoglobulin G deposition in lungs might play a role in the development of this reaction. Our patient might already be sensitized to other sulfonamides.

There are several possible explanations for the lack of clarity regarding the risk of respiratory failure among critically ill patients. First, this adverse effect is very rare, and many doctors may not consider the involvement of acetazolamide at the first episode. Although two case reports^4^, ^5^ have identified episodes of anaphylaxis and pulmonary edema, they did not present anaphylaxis‐specific symptoms like skin erythema. Moreover, Zimmermann *et al*.^2^ suggested that this mechanism was not connected to an allergic reaction, as they could not identify a specific antigen or allergic reaction to acetazolamide during the skin prick test. Second, pulmonary edema is fairly common in intensive care units, and it might be missed or attributed to another pathophysiology. For example, we considered that the first episode in our patient was a different problem because of the clinical course. In contrast, the reported cases have involved stable patients preparing for scheduled procedures. Thus, the emergence of pulmonary edema might be considered strange and worthy of investigation. Finally, although acetazolamide is frequently chosen as a respiratory stimulant and diuretic, We chose the drug twice before noticing the connection between acetazolamide treatment and respiratory deterioration.

## Conclusion

In the present case, we encountered rare and potentially fatal episodes of pulmonary edema that was provoked by treatment with acetazolamide. Based on both our experience with this adverse event and the fatal outcomes in some reported cases, we believe that careful attention should be paid when treating critically ill patients with acetazolamide.

## Conflict of interest

None declared.
